# Predictors of cardiopulmonary arrest outcome in a comprehensive cancer center intensive care unit

**DOI:** 10.1186/1757-7241-21-18

**Published:** 2013-03-20

**Authors:** Faisal A Khasawneh, Mahmoud T Kamel, Mohammad I Abu-Zaid

**Affiliations:** 1 Section of Critical Care Medicine, Department of Internal Medicine, School of Medicine, Texas Tech University Health Sciences Center, 1400 S. Coulter Street, Amarillo, Texas, 79106, USA; 2 Department of Internal Medicine, School of Medicine, University of Arizona, Tucson, AZ, USA

**Keywords:** Cardiopulmonary arrest, Critically-ill cancer patients, Outcome predictors

## Abstract

**Background:**

Some comprehensive cancer centers in industrialized countries have reported improved outcomes in their cardiopulmonary arrest (CPA) patients. Little is known about the outcomes and predictors of CPA in cancer centers in other parts of the world. The objective of this study was to examine the predictors of CPA outcome in a comprehensive cancer center closed medical-surgical intensive care unit (ICU) located in Amman, Jordan.

**Methods:**

In this retrospective single-center cohort study, we identified 104 patients who had a CPA during their stay in the ICU between 1/1/2008 and 6/30/2009. Demographic data and CPA-related variables and outcome were extracted from medical records. Comparisons between different variables and CPA outcome were conducted using logistic regression.

**Results:**

The mean age of the group was 49.7 ± 15.3 years. The mean APACHE II score was 23.7 ± 8.0. Thirty six patients (34.6%) were resuscitated successfully but 8 of them (7.7% of the cohort) left the ICU alive and only 6 out of the 8 (5.8% of the cohort) left the hospital alive. The following variables predict resuscitation failure: acute kidney injury (OR 1.7, CI: 1.1 – 2.6), being on mechanical ventilation (OR 3.8, CI: 1.3 – 11), refractory shock (OR 4.7, CI: 1.8 – 12) and CPR duration (OR 1.1, CI: 1.1 – 1.2).

**Conclusion:**

Survival among cancer patients who develop CPA in the ICU continues to be poor. Once cancer patients suffered a CPA in the ICU multiple factors predicted resuscitation failure but CPR duration was the only factor that predicted resuscitation failure and ICU as well as hospital mortality.

## Introduction

Malignancy has been identified as a poor prognostic indicator for in-hospital cardiopulmonary arrest (CPA) [1,2]. Furthermore, in cancer patients with advanced disease, long-term benefits of cardiopulmonary resuscitation (CPR) are limited, even if they survive the initial CPA [3]. This is due to the progressive nature of the disease precluding long-term survival and diminishing vital organs’ functional reserve. Some comprehensive cancer centers in developed countries have reported improved CPA outcomes [4,5]. This improvement is attributed to advances in critical care medicine and candid discussions between cancer patients and their treating physicians which allow some of those patients to elect not to be resuscitated in the event of a CPA.

Data regarding the outcomes of critically ill cancer patients in developing countries, especially from the Middle East, is scare. Thus, the aim of this study was to examine the outcomes of CPA in critically ill cancer patients in a closed medical-surgical intensive care unit (ICU) in a comprehensive cancer center in Amman, Jordan. It also identified the predictors of successful CPR, and ICU and hospital outcomes.

## Methods

The charts of adult patients who had CPA while hospitalized in the closed medical-surgical ICU of King Hussein Cancer Center in Amman, Jordan between 1/1/2008 and 6/30/2009 were retrospectively reviewed. King Hussein Cancer Center is a 120-bed comprehensive cancer center with 11-bed ICU [6]. It is a Joint Commission International accredited medical center since 2006 and its laboratory is a College of American Pathologists’ accredited lab since 2009. The institutional review board at King Hussein Cancer Center approved the study protocol, approval number: 08KHCC37.

### Definitions

CPA was defined as having all of the following: 1) no palpable pulse, 2) no detectable blood pressure, 3) no effective respiration or loss of breath triggering in mechanically ventilated patients, and 4) loss of consciousness in previously awake patients attributable only to loss of adequate brain perfusion [4]. Patients suffering isolated respiratory arrest requiring only assisted ventilation without loss of a palpable pulse necessitating external cardiac message were excluded. Patients who had a CPA outside the ICU were excluded, too. In patients with more than one arrest during the same hospital stay, we included only the first CPA.

Acute kidney injury was defined as an abrupt (within 48 hours) absolute increase in serum creatinine ≥ 26.4 micro moles/liter, a percentage increase in serum creatinine of ≥ 50%, or a urine output of < 0.5 milliliter/kilogram/hour for 6 hours [7]. Refractory shock was defined as requiring 2 or more vasopressors to maintain a mean arterial pressure of above 65. Acute respiratory failure was defined as the need for fraction of inspired oxygen of 50% or more and/or positive pressure mechanical ventilation to maintain an oxygen partial pressure (PO_2_) of above 55 and a carbon dioxide partial pressure (PCO_2_) of below 50. Patients who failed to meet the above PO_2_ and PCO_2_ goals despite of 100% oxygen supplementation and optimal conventional mechanical ventilation were considered to be in refractory respiratory failure. Patients who had an arrest due to refractory shock, refractory respiratory failure and/or multiorgan dysfunction syndrome were labeled to have had an anticipated CPA.

### Data collection

The charts that met the above criteria were reviewed for the following pre-arrest variables; age, gender, primary site of malignancy and its progression, reason for ICU admission, Acute Physiology and Chronic Health Evaluation II (APACHE II) score, CPA cause, prior code status discussions and the need for mechanical ventilation, vasopressors use and hemodialysis. The level of arterial oxygenation and acidemia prior to the CPA was not documented.

Whenever a patient had had a CPA, the patient’s nurse activated a bedside automated house-wide alarm and initiated CPR. An advanced cardiac live support-trained team responded and provided care meanwhile an arrest record is generated and subsequently placed in that patient’s chart. The above mentioned team consisted of senior internal medicine and surgery residents, a respiratory therapist, a pharmacist and the hospital’s nursing supervisor. The patient received 1 milligram of epinephrine every 3 to 5 minutes. Atropine, amiodarone and defibrillation were provided as recommended by the American Heart Association guidelines. The already running vasopressors were not titrated during the CPA. Running antiarrhythmics were stopped if the treating physicians felt they are contributing to the arrest, for example beta blockers or amiodarone causing the antecedent bradycardia or hypotension. In addition to the above, efforts were focused on identifying potentially reversible causes like hyperkalemia, hypovolemia, tension pneumothorax, cardiac tamponade and acidemia.

The arrest records were reviewed and the following arrest-related variables were noted; initial rhythm being one of the following; pulseless electrical activity (PEA), asystole, ventricular fibrillation or pulseless ventricular tachycardia; duration of CPA in minutes; and the outcome of CPA. The duration of CPA was determined from the arrest record and was defined as the time from initiating that event recording to either return of spontaneous circulation or cessation of resuscitation efforts. The time from CPA to the arrival of advanced cardiac live support-trained team could not be documented. In addition, ICU and hospital mortality and length of stay (LOS) prior to and after CPA (until death or hospital discharge) were recorded. Therapeutic hypothermia was not practiced in the cancer center during the study period.

### Statistical analysis

For continuous variables, the mean with standard deviation or the median with a range were reported. For categorical variables, the number of patients with that variable and a corresponding percentage were reported. Univariate comparisons between alive and deceased patient groups corresponding to the above collected variables were performed by logistic regression. The results were expressed as odds ratio (OR) with confidence interval (CI) or *p*-values. A *p*-value of less than 0.05 was considered to be statistically significant. Forward stepwise logistic regression was used to construct a model from the significant variables identified by univariate analysis. The statistical analysis was done using Stata 12 (StataCorp LP, College station, Texas).

## Results

During the study period, 761 patients were admitted to the ICU and128 cardiopulmonary arrests occurred in 104 patients. Twenty-four events occurred as second or third CPA and were excluded. Period prevalence of CPA was 13.7%. The most common cause for ICU admission in the cohort was severe sepsis or septic shock, diagnosed in 39 patients (37.5%), and followed by acute respiratory failure which accounted for 27 admissions (26.0%). The study population is summarized in Table 1.

**Table 1:** The study population characteristics

**Characteristic**	**Number**
Patients suffered CPA	104
Mean age (years ± SD)	49.7 ± 15.3
Gender:	
Men	65
Women	39
APACHE II (score ± SD)	23.7 ± 8.0
Patients on MV at the time of CPA	86 (82.7%)
Patients on vasopressors at the time of CPA	77 (74.0%)
Patients on HD at the time of CPA	15 (14.4%)
Malignancies:	
Solid tumors	57 (54.8%)
Hematologic malignancies	47 (45.2%)

APACHE II: Acute Physiology and Chronic Health Evaluation II; CPA: cardiopulmonary arrest; HD: hemodialysis; MV: mechanical ventilation; SD: standard deviation.

Among patients with hematologic malignancy; 28 (26.9%) had leukemia, 15 (14.4%) had lymphoma and 4 (3.9%) had multiple myeloma. Among patients with solid tumors, lung cancers and gastrointestinal cancers accounted for 14 cases (13.5%) each. There were 7 patients (6.7%) with breast cancer and 6 patients (5.8%) with genitourinary cancers. A variety of other solid tumors including sarcomas, mesotheliomas and head and neck tumors accounted for the 16 remaining cases (15.4%). Forty nine patients (47.1%) had a progressing or a relapsed cancer. Eleven patients (10.6%) had a newly diagnosed cancer, 7 patients (6.7%) had a regressing tumor, one patient (0.96%) had a stable disease and in another the cancer was in remission. Malignancy stage and progression were not documented in 35 cases (33.7%). Forty-eight patients (46.2%) had acute kidney injury.

The mean age of patients with hematological malignancies was 45.1 ± 14.9 years *vs.* 53.2 ± 14.7 years in patients with solid tumors (*p* = 0.006). Mechanical ventilation and vasopressors were required in 93.3% and 86.7% of patients with hematological malignancies compared with 74.6% and 64.4% in patients with solid tumors (*P* = 0.0172 and 0.0129, respectively). The difference between the 2 groups mean APACHE II score was not statistically significant (data not shown).

The code status “the patient’s wishes to undergo CPR in the event of a CPA” was discussed in 12 cases (11.5%). The mean time from ICU admission to CPA was 4.4 ± 6.9 days. The most commonly recorded initial rhythm was asystole (59 patients, 56.7%) followed by PEA (43 patients, 41.4%). Two patients (1.9%) had pulseless ventricular tachycardia. Shock and acute respiratory failure were the most common causes of CPA accounting for 69 (66.3%) and 18 (17.3%) of the CPAs, respectively. The mean duration of CPR was 17.9 ± 14.6 minutes. Thirty six patients (34.6%) were resuscitated successfully but 8 of them (7.7% of the cohort) left the ICU alive and only 6 out of the 8 (5.8% of the cohort) left the hospital alive. The average ICU and hospital LOS were 5.6 ± 7.1 days and 10.4 ± 9.9 days, respectively.

Univariate comparisons between alive and deceased patient groups corresponding to the above collected variables are shown in Table 2. On multivariate analysis, CPR duration was the only variable that had statistically significant association with outcomes of interest; CPR outcome, 1.12 (1.05 – 1.19), ICU outcome, 1.13 (1.00 – 1.28), hospital outcome, 1.35 (1.06 – 1.72). This translates to a 10% increase in mortality per minute of resuscitation.

**Table 2:** Univariate comparisons between alive and deceased cardiopulmonary arrest patient groups

**Variable**	**CPR failure OR(CI)**	**ICU death OR(CI)**	**Hospital death OR(CI)**
Age	0.99 (0.97–1.02)	0.99 (0.94–1.04)	0.98 (0.93–1.04)
Gender	1.6 (0.68–3.6)	1.7 (0.41–7.4)	0.82 (0.14–4.7)
Hematologic malignancies	0.39 (0.17–0.92)	No estimate	No estimate
Progressing or relapsed tumor	0.74 (0.24–2.3)	4.1 ( 0.64–27)	5.3 (0.46–62)
Acute kidney injury *	1.7 (1.1–2.6)	1.7 (0.65–4.7)	6.6 (0.56–79)
Being on MV	3.8 (1.3–11.0)	3.2 (0.70–15)	5.5 (1.0–30)
Refractory shock**	4.7 (1.8–12.0)	6.4 (0.75–50)	5.7 (0.34–97)
Lactate ≥ 4	1.1 (0.95–1.2)	1.4 (0.82–2.5)	1.7 (0.67–4.5)
ICU LOS before CPA	1.00 (0.99–1.00)	1.01 (0.99–1.02)	1.17 (0.88–1.58)
Anticipated CPA***	1.6 (0.68–3.8)	14 (1.6–121)	8.6 (0.93–81)
Length of CPR	1.1 (1.1–1.2)	1.1 (1.0–1.3)	1.3 (1.1–1.7)

CI: confidence interval; CPA: cardiopulmonary arrest; CPR: cardiopulmonary resuscitation; ICU: intensive care unit; LOS: length of stay; MV: mechanical ventilation; OR: odds ratio.

*Acute kidney injury: the abrupt (within 48 hours) absolute increase in serum creatinine ≥ 26.4 micro moles/L, a percentage increase in serum creatinine of ≥ 50%, or a urine output of < 0.5 milliliter/kilogram/hour for 6 hours.

**Refractory shock: requiring 2 or more vasopressors to maintain a mean arterial pressure of above 65.

***Anticipated CPA: cardiopulmonary arrest in patients with refractory shock, refractory respiratory failure and/or multiorgan dysfunction syndrome.

## Discussion

Despite multiple advances in critical care medicine and cardiac life support, cancer patients’ survival following CPA in the ICU remains poor [2]. In our study, CPA outcome predictors in 104 critically ill cancer patients in a comprehensive cancer center were evaluated. About one third of the cohort (34.6%) survived the initial CPA but only 1 in 20 patients (5.8%) survived to hospital discharge. Developing acute kidney injury or refractory shock, being on mechanical ventilation and CPR duration predicted resuscitation failure meanwhile requiring mechanical ventilation and CPR duration predicted poor hospital outcome. Cancer patients who suffered CPA in the ICU had a 10% increase in mortality per minute of resuscitation.

A meta-analysis of 42 studies, the vast majority of which were carried-out in developed countries, examined survival in 1707 cancer patients who underwent in-hospital CPR reported an overall hospital survival of 6.2% (95% CI: 3.2 – 9.1) [8]. ICU survival rate in the pooled data was 2.2% (95% CI: 0 – 4.6), findings that are consistent with ours.

Predictors of in-hospital CPA outcome were reported by several investigators [1,3,9-14]. Survival was significantly low in unwitnessed arrests, in anticipated arrests, after PEA/asystole arrests, and if the resuscitation efforts lasted longer than 10 minutes. Furthermore, in cancer patients, poor functional status at baseline, metastatic disease and hematological malignancies carried grim prognosis [8,15]. As an initial cardiac rhythm, all but two of our study subjects had PEA or asystole which precluded further testing of initial rhythm’s effect on outcome. Cardiac arrests in our study developed in the ICU setting, hence, were all witnessed. There is no agreed upon definition for “anticipated CPA”. Anticipated CPA defined as having refractory shock, refractory respiratory failure and/or multiorgan dysfunction syndrome did not predict CPR failure or hospital outcome. In line with previous studies, our data could not identify a significant relation between age and CPA outcome. In our data, CPR duration had a strong association with outcome and it has been previously used in the derivation of a clinical decision rule to discontinue resuscitation efforts in CPA inpatients [9]. The rule included initial cardiac rhythm and whether the arrest was witnessed.

Patients with hematological malignancies are less likely to survive a CPA compared with patients with solid tumors [8]. In our study, patients with hematological malignancies were more likely to survive a first CPA but none survived to hospital discharge. In comparison to patients with solid tumors, those with hematological malignancies were relatively younger but were more likely to be on mechanical ventilation and more likely to require vasopressor support.

Culture and religion are among a multitude of factors that influence end-of-life decisions [16]. They impact the perception and behavior of patients and their treating physicians [17]. In Jordan, like it is the case in the Middle East and some Southern and Eastern European countries, the principles of beneficence and non-malfeasance outweigh patient autonomy and play a predominant role in the process of decision making [16]. Moreover, illness in some of those countries is considered to be a shared family affair complicating end-of-life-discussions furthermore [18].

Apropos to the above, code status discussions were documented in 11% of our patients, a small percentage in light of how critically ill the cohort was; 82% on mechanical ventilation and 74% on vasopressors. The development of palliative care medicine has made wide strides in Jordan but, for the reasons alluded to above, end of life discussions remains a sensitive topic [19]. Although it is easy for patients and families to understand the “do-not-resuscitate” (DNR) concept, they find it a very difficult choice to accept. Cultural beliefs and some religious misconceptions stand behind this sense of guilt and discomfort. To overcome this, some physicians have resorted to their legal and religious background and do not present DNR status as a choice, rather a medical decision that the family is informed of [19]. With globalization and as the number of Western-trained physicians increase, a change in how end-of-life-discussion is handled in this part of the world is to be expected. In fact, this has already been reported by some of the most recent publications [20].

The present study has several limitations. Its retrospective nature introduced a selection bias by including patients who underwent CPR, but not those who had a CPA but were not resuscitated because of a DNR code status. Due to lack of testing and adequate documentation, the study did not include some of the well described predictors of CPA survival like end-tidal carbon dioxide levels during CPR and baseline functional status of patients. The small number of cases in a single-center and the specificity of the patient population studied “critically ill cancer patients” are other limitations too; this prevents generalizability and requires further validation.

## Conclusions

The outcome of CPA in critically ill cancer patients continues to be poor. After an open discussion with the patient and family and using good clinical judgment, CPA outcome predictors described in this study and previous literature should be used to guide CPR efforts. The intention is to perform CPR meanwhile trying to treat a reversible disease process. CPR prolonging inevitable death should be discouraged. This will decrease time and efforts spent in futile care, shorten families’ hopeless waiting and lead to significant cost savings.

## Consent

Written informed consent from patients was waived because of the retrospective nature of the study.

## Competing interest

The authors declare that they have no competing interests

## Authors’ contributions

FAK, MTK, MIA reviewed the patients' charts and collected the data. FAK wrote the initial manuscript draft. MTK and MIA read and audited the manuscript. All authors read and approved the final manuscript.

## Authors’ information

The work was done at King Hussein Cancer Center, Amman, Jordan.
